# Strategies and tactics to perform safe pancreaticoduodenectomy for 94-year-old patient: report of a case

**DOI:** 10.1186/s40792-022-01395-9

**Published:** 2022-03-04

**Authors:** Yu Suyama, Koichiro Haruki, Ryoga Hamura, Masashi Tsunematsu, Yoshihiro Shirai, Tomohiko Taniai, Mitsuru Yanagaki, Kenei Furukawa, Shinji Onda, Hiroaki Shiba, Toru Ikegami

**Affiliations:** Division of Hepatobiliary and Pancreatic Surgery, Department of Surgery, The Jikei University School of Medicine, 3-25-8, Nishi-Shinbashi, Minato-ku, Tokyo, 105-8461 Japan

**Keywords:** Pancreaticoduodenectomy, elderly, sarcopenia, osteopenia

## Abstract

**Background:**

Despite improvement of postoperative management, pancreatoduodenectomy still has a high rate of major complications. Therefore, careful assessment is critically important when we consider high risk surgery for extremely elderly patients.

**Case presentation:**

A 94-year-old man, who suffered dark urine, epigastric pain, and loss of appetite, was diagnosed as bile duct cancer and underwent endoscopic retrograde biliary drainage. He has past history of hypertension and paroxysmal atrial fibrillation. Computed tomography (CT) showed a nodule in the lower bile duct, which was slowly enhanced by dynamic CT. The patient was evaluated whether he overcomes pancreatoduodenectomy by cardiac ultrasonography, brain magnetic resonance angiography, nutritional evaluation (rapid turnover proteins), and CT-based general assessment, including sarcopenia and osteopenia. The patient was independent in activities of daily living and has enough ejection fraction of 65%, and examinations revealed no impairment of cognitive function, sarcopenia, and osteopenia. With a diagnosis of bile duct cancer with no distant metastasis, the patient underwent subtotal stomach-preserving pancreatoduodenectomy with lymph node dissection. Operation time was 299 min and estimated blood loss was 100 ml. Pathological examination revealed papillary adenocarcinoma of the bile duct (pT3N1M0 Stage IIIB). Enteral nutrition was given through jejunostomy and then the patient started oral intake after an evaluation of swallowing function. Postoperative course was uneventful and all drains including pancreatic duct stent, biliary stent, and jejunostomy were removed by 3 weeks after operation. The levels of rapid turnover proteins dropped at postoperative day 7, but recovered at 1 month after operation via appropriate nutrition and rehabilitation. He remains well with no evidence of tumor recurrence as of 1 year after resection.

**Conclusions:**

We herein report successfully treated cases of bile duct cancer in 94-year-old patient by pancreatoduodenectomy with careful evaluation of osteopenia, sarcopenia and nutrition.

## Background

With rapid increase of the proportion of the elderly population, there will be an increasing need to consider patients over the age of 80 for pancreaticoduodenectomy (PD) for the treatment of biliary and pancreatic cancer [1]. However, despite improvement of postoperative management, PD still has a high rate of major complications, including pancreatic fistula, bleeding, intra-abdominal collection, and pulmonary complications, especially in elderly patients [2]. Moreover, frailty including sarcopenia and osteopenia has been associated with high risk of postoperative complications and morbidity [1, 3–5]. Therefore, careful assessment is critically important when we consider high risk surgery for elderly patients.

We herein report successfully treated cases of bile duct cancer in 94-year-old patient by pancreatoduodenectomy after detailed general assessment, including nutritional and general condition. To best of our knowledge, this is the oldest patient who underwent PD in the English literature.

## Case presentation

**Fig. 1:**
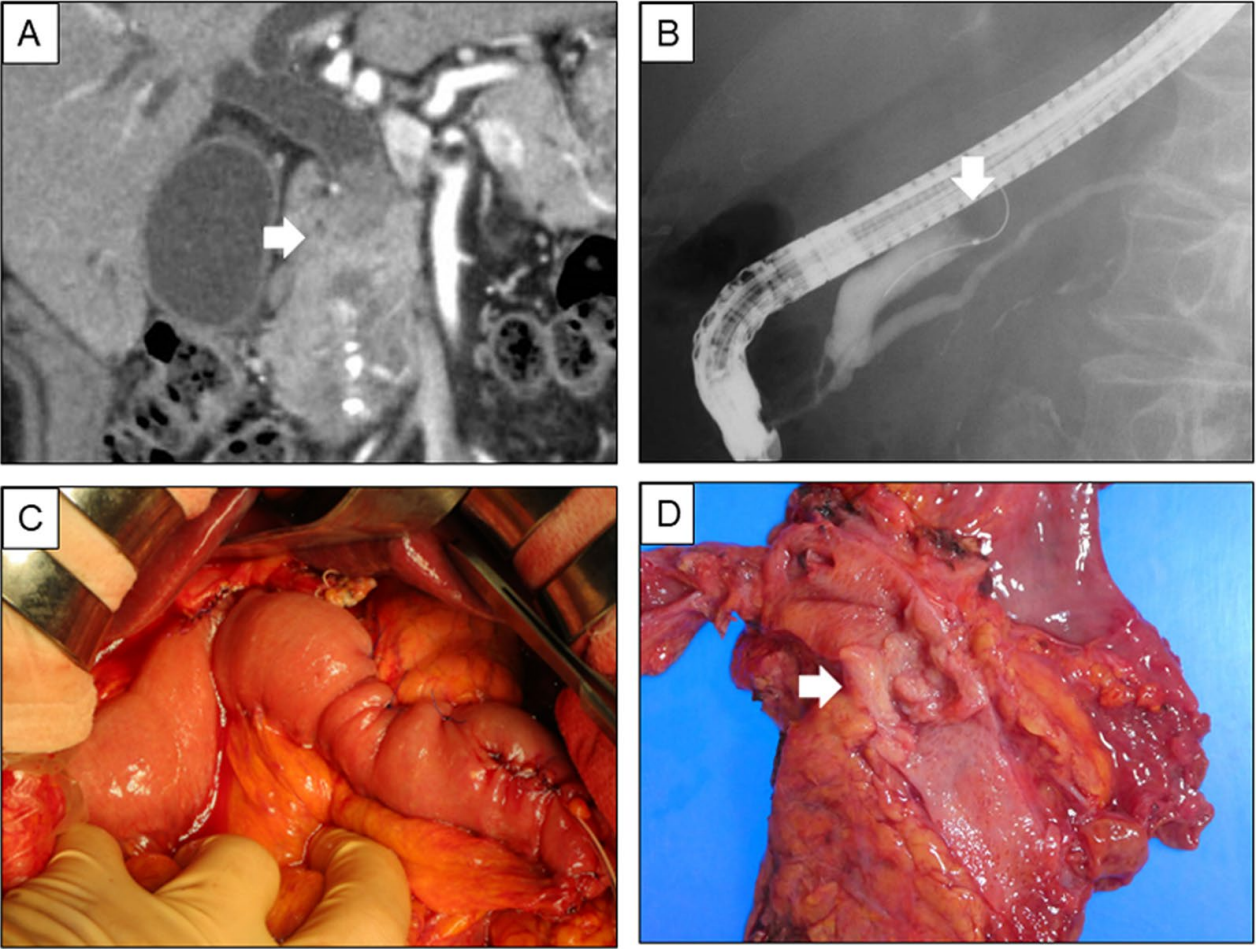
Imaging of bile duct tumor. Computed tomography showed a nodule in the lower bile duct, which was slowly enhanced by dynamic CT (**A**, arrow). Endoscopic retrograde cholangiopancreatography revealed biliary obstruction (**B**, arrow). Pancreaticoduodenectomy with lymph node dissection (**C**). The resected specimens showed a tumor in the lower bile duct (**D**, arrow)

**Fig. 2:**
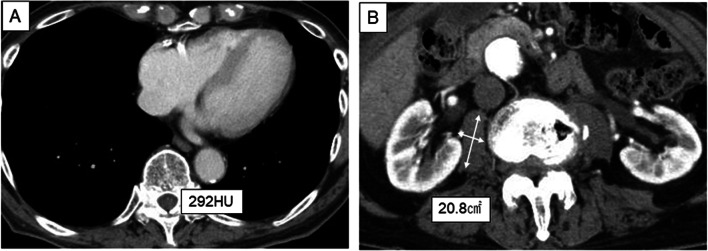
Evaluation of osteopenia and sarcopenia. Osteopenia was evaluated by bone mineral density calculated using average od pixel density in the midvertebral core of the 11th thoracic vertebra and defined according to the standard bone mineral density (**A**). Sarcopenia was evaluated by psoas muscle areas at the third lumber vertebra and defined according to the sex-specific average (**B**)

A 94-year-old man, who suffered dark urine, epigastric pain, and loss of appetite, was admitted for evaluation and treatment of bile duct cancer. He has a past history of hypertension and paroxysmal atrial fibrillation. Computed tomography (CT) showed a nodule in the lower bile duct, which was slowly enhanced by dynamic CT (Fig. 1A). Tumor markers were as follows: carcinoembryonic antigen of 7.2 ng/mL, carbohydrate antigen 19–9 of 22 U/mL, respectively. Endoscopic retrograde cholangiopancreatography revealed biliary obstruction and biliary drainage was performed (Fig. 1B). Cytopathological examination revealed adenocarcinoma of the bile duct. The patient was carefully evaluated whether he overcame pancreatoduodenectomy by cardiac ultrasonography, brain magnetic resonance angiography, nutritional evaluation by rapid turnover proteins (RTPs) (retinol-binding protein of 2.4 mg/dL, pre-albumin of 19.9 mg/dL, transferrin of 196 mg/dL), and CT-based assessment, including osteopenia and sarcopenia. Sarcopenia was evaluated by the area of the psoas muscle at the caudal end of the third lumbar vertebra by measurement of the lengths of the major and minor axes of the psoas muscle [6]. We then evaluated sarcopenia by comparing the area of psoas muscle with previously reported sex-specific average [7]. Osteopenia was defined as actual bone mineral density (BMD) below the calculated standard BMD, which was calculated as previously reported (308.82–2.49 × age in men and 311.84–2.41 × age in women) [5]. BMD was measured in trabecular bone by calculating average pixel density within a circle in midvertebral core at the bottom of 11th thoracic vertebra (Th11) on preoperative computed tomography. The patient was independent in activities of daily living and was graded as performance status 1 and American Society of Anesthesiologists physical status (ASA-PS) 2. He had enough ejection fraction of 65%, and examinations revealed no impairment of cognitive function and neither of osteopenia (Fig. 2A) and sarcopenia (Fig. 2B).

**Fig. 3:**
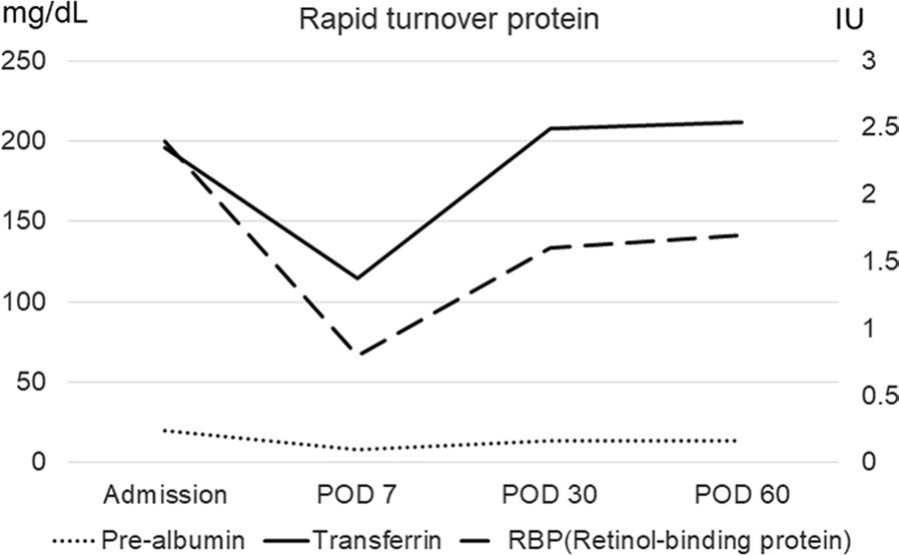
Assessment of rapid turnover proteins. Rapid turnover proteins including retinol-binding protein, pre-albumin, and transferrin were measured preoperatively and postoperative days 7, 30, and 60

With a diagnosis of bile duct cancer with no distant metastasis, the patient underwent subtotal stomach-preserving pancreaticoduodenectomy with lymph node dissection (Fig. 1C). Operation time was 299 min and estimated blood loss was 100 ml. Pancreatic duct stent (6Fr), external biliary stent (7.5Fr), jejunostomy, and 2 drain tubes (Winslow and anastomosis of pancreatojejunostomy) were placed after operation. The resected specimens showed a tumor in the lower bile duct (Fig. 1D). Pathological examination revealed papillary adenocarcinoma of the bile duct (pT3N1M0 Stage IIIB) and the surgical margin was negative. Enteral nutrition (ENEVO®, Abbott, Japan) was given through jejunostomy and then the patient started oral intake after an evaluation of swallowing function on the postoperative day 7. Although RTPs were decreased on the postoperative day 7 (retinol-binding protein of 0.8 mg/dL, pre-albumin of 8.1 mg/dL, transferrin of 115 mg/dL), those were recovered after 1 month (retinol-binding protein of 1.6 mg/dL, pre-albumin of 13.7 mg/dL, transferrin of 208 mg/dL) (Fig. 3) with careful nutritional support and rehabilitation. The patient discharged on postoperative day 65 without any complications. He remains well with no evidence of tumor recurrence as of 1 year after resection.

## Discussion

Morbidity and mortality of PD have improved recently with improvement in intensive care management and surgical techniques. However, the prevalence of postoperative complications including a 30-day morbidity rate and morbidity rate is not satisfactory in elderly and patients with frailty [1]. There have been an increasing number of publications over the past decade reporting on the outcomes of PD in patients aged 80 years and older compared to younger patients [8]. In elderly patients, the morbidity associated with PD related to surgical specific and non-specific complications remains high. The patients aged 80 years or older were 1.5-fold more likely to have complications than their younger counterparts. Especially, the rate of pneumonia was significantly higher in the older patient group [1, 8]. In contrary, several studies reported that age was not an independent risk factor for perioperative mortality and morbidity following PD and suggested that patients should not be excluded from PD due to age [9]. Therefore, preoperative assessment beyond the age might be important to predict postoperative complications. In the current case, although the patient’s age was 94 years, he was graded as performance status 1 and ASA-PS of 2, and was independent in activities of daily living. Thus, we could have considered to perform PD for this patient.

Studies indicated that delirium and pneumonia, which are specific complications of elderly patients, may prolong hospital stay and have negative impact on patient’s recovery after operation [1, 10]. Evidence suggests that nursing protocols including orienting communication, oral and nutritional assistance, and early mobilization can reduce postoperative delirium after abdominal surgery [11]. Considering the high risk of delirium in this patient, we proceeded with early rehabilitation and frequent communication to prevent postoperative delirium after PD. In addition, given that pneumonia can have possibility to cause mortality in this patient, we prolonged his oral intake and administered enteral nutrition via jejunostomy to prevent bacterial translocation before careful evaluation of swallowing function. Although routine jejunostomy is not recommended for the patients who can start oral intake [12], temporally enteral nutrition may be useful for such case who cannot start oral intake immediately.

Recent evidence suggests that sarcopenia and osteopenia has been associated with postoperative complications as well as long-term outcomes [1, 3, 8]. Sarcopenia, defined was the loss of skeletal muscle mass, has been frequently observed in elderly patients, which is the result of an imbalance between protein and degradation. Osteopenia, defined as loss of bone density, has been reported as an early marker of deconditioning that precedes sarcopenia [5]. Therefore, assessment of sarcopenia and osteopenia in elderly patients would be useful to evaluate surgical risk for PD. In the present case, the patient did not show either of sarcopenia and osteopenia. Moreover, studies have shown the negative impact of sarcopenia and osteopenia on survival in several malignancies [5, 6]. Preoperative rehabilitation, including resistance exercise and aerobic exercise, can improve these muscle and bone status, and which may reduce postoperative complication [13] and can potentially contribute to prolonged survival in cancer patient. Although we administered enteral nutrition in this patient, immunonutrition has been reported to reduce infectious complication [14].

RTPs, such as prealbumin, transferrin, and retinol-binding protein are important for the assessment of nutritional status in cancer patients [15]. The measurement of serum concentrations of each RTP has been reported to be more accurate than albumin in the assessment of nutritional status. [16] Since RTPs have short half-lives, RTPs can provide dynamic nutritional assessment and can be improved by nutritional intervention including perioperative nutritional support. In the present case, we evaluated these proteins and observed that these proteins recovered on postoperative day 30 with appropriate nutritional support by enterally and orally.

## Conclusions

We successfully performed PD for a 94-year-old Japanese man with careful evaluation of osteopenia, sarcopenia and nutrition.

## Data Availability

All data generated or analyzed during this study are included in this published article.

## Abbreviations

ASA-PS: American Society of Anesthesiologists physical status
CT: Computed tomography
PD: Pancreaticoduodenectomy
RTP: Rapid turnover protein
